# Alpha-mangostin decreases high glucose-induced damage on human umbilical vein endothelial cells by increasing autophagic protein expression

**DOI:** 10.22038/IJBMS.2023.71019.15425

**Published:** 2024

**Authors:** Farhad Eisvand, Kasra Rezvani, Hossein Hosseinzadeh, Bibi Marjan Razavi

**Affiliations:** 1 Department of Pharmacodynamics and Toxicology, School of Pharmacy, Mashhad University of Medical Sciences, Mashhad, Iran; 2 Pharmaceutical Research Center, Pharmaceutical Technology Institute, Mashhad University of Medical Sciences, Mashhad, Iran; 3 Targeted Drug Delivery Research Center, Pharmaceutical Technology Institute, Mashhad University of Medical Sciences, Mashhad, Iran

**Keywords:** Alpha-mangostin, Autophagy, Beclin 1, Diabetes, Garcinia mangostana, HUVEC, LC3, SIRT1

## Abstract

**Objective(s):**

Diabetes is a chronic disorder that occurs as a result of impaired glucose metabolism. In hyperglycaemic states, the balance between oxidative stress and antioxidant enzymes is disrupted leading to oxidative damage and cell death. In addition, impaired autophagy leads to the storage of dysfunctional proteins and cellular organelles in the cell. Hence, the cytoprotective function of autophagy may be disrupted by high glucose conditions. Alpha-mangostin (A-MG) is an essential xanthone purified from the mangosteen fruit. The different pharmacological benefits of alpha-mangostin, including antioxidant, anti-obesity, and antidiabetic, were demonstrated.

**Materials and Methods:**

We evaluated the protective influence of A-MG on autophagic response impaired by high concentrations of glucose in human umbilical vein endothelial cells (HUVECs). The HUVECs were treated with various glucose concentrations (5-60 mM) and A-MG (1.25-10 μM) for three days. Then, HUVECs were treated with 60 mM of glucose+2.5 μM of A-MG to measure viability, ROS, and NO content. Finally, the levels of autophagic proteins including LC3, SIRT1, and beclin 1 were evaluated by western blot.

**Results:**

The results expressed that high glucose condition (60 mM) decreased viability and increased ROS and NO content in HUVECs. In addition, LC3, SIRT1, and beclin 1 protein levels declined when HUVECs were exposed to the high concentration of glucose. A-MG reversed these detrimental effects and elevated autophagic protein levels.

**Conclusion:**

Our data represent that A-MG protects HUVECs against high glucose conditions by decreasing ROS and NO generation as well as increasing the expression of autophagy proteins.

## Introduction

Diabetes is a chronic disease that occurs as a result of impaired glucose metabolism and is characterized by high levels of glucose in the blood (1, 2). Because of some factors such as physical inactivity, aging, population increase, urbanization, and obesity, the number of persons with diabetes is growing. The problems of diabetes comprise macrovascular (stroke, peripheral vascular disease, and ischemic heart disease) and microvascular (neuropathy, nephropathy, and retinopathy) endpoints (1). The estimated number of persons with diabetes will elevate from 171 to 366 million between 2000 and 2030 (3).

Aged and injured cells or organelles are cleared by two processes apoptosis and autophagy which depending on the threshold can induce either one (4, 5). Autophagy is a catabolic procedure that involves the sequestration and degradation of cell organelles and proteins by lysosomal machinery and balances the degradation, synthesis, and next recycling of cellular ingredients (6, 7). Autophagosome formation is induced by several genes comprising beclin-1, LC3, and ATGs (8). Beclin 1 is a class III phosphoinositide 3-kinase and was separated as a Bcl-2-interacting protein that suppresses cancer cells in mammalian systems (9). LC3 is the main regulator of autophagy which starts autophagosome generation after conjugating with ATG7 and other autophagic agents (10). On the other hand, a key anti-aging regulator is SIRT1 which controls the proteins related to cell survival pathways, metabolism, and oxidative. This protein is reduced in old vascular tissue which leads to inflammation, atherosclerosis, excessive oxidative stress, and arterial aging (11, 12).

High glucose condition upsets the balance between oxidative stress and antioxidant capacity and leads to increased oxidative stress and cell damage. Autophagy machinery removes damaged substrates in the cell, the high glucose condition interferes with this function (13, 14).

Several natural products including crocin (15),* Nigella sativa* oil (16), quercetin (17), and resveratrol (18) have shown anti-diabetes effects. Mangosteen (*Garcinia mangostana*) Linn is a tropical tree that is cultivated in Southeast Asia including Myanmar, Thailand, India, Philippines, Malaysia, and Sri Lanka (19). In the 1950s, a yellowish color matter was extracted among the essential xanthones purified from the mangosteen fruit that is called alpha-mangostin (A-MG) (20). *In vitro* and *in vivo* investigations reported that A-MG has different pharmacological benefits including anti-inflammatory, antioxidant, antibacterial, anti-obesity, antidiabetic, and anti-bacterial activities along with hepatoprotective, cardioprotective, and neuroprotective features (21-24). It was reported that A-MG has protective effects on high-glucose-induced apoptosis via decreasing apoptotic proteins including cleaved caspase-3 and Bax (25).

Another study reported that A-MG reduced ROS formation and inhibited high-glucose-induced apoptosis by the JNK / MAPK pathway in endothelial cells (26). In the current investigation, we evaluated the protective effects of A-MG on high glucose-induced damage on HUVEC by enhancing autophagy.

## Materials and Methods

***Materials***

Alpha-mangostin (Trademax Pharmaceuticals & Chemicals Co, China), Dulbecco’s modified Eagles Medium‐F12 (DMEM‐ F12), penicillin-streptomycin solution and trypsin (Bioidea, Iran), Fetal bovine serum (Gibco, USA), 3- (4,5-dimethyl-2-thiazolyl)-2,5-diphenyl-2Htetrazolium bromide (MTT; AtoCell, Budapest), BSA (Solarbio, China), ethylene glycol tetraacetic acid (EGTA; Sigma, USA), dry skim milk (Quetlab, UK), NaF (Sigma, USA), ethylenediaminetetraacetic acid (EDTA; Pars Tous Biotechnology, Mashhad, Iran), sodium orthovanadate (Na_3_ VO_4_; Sigma, Madhya Pradesh, India), sodium deoxycholate (Sigma, New Zealand), Protease and phosphatase inhibitor cocktail (Thermo Fisher Scientific, USA). Polyvinylidene difluoride (PVDF) and Protein assay kit (Bradford reagent) were purchased from Bio-Rad, USA. 

***Cell culture***

HUVEC cells were purchased from the Pasteur Institute (Tehran, Iran) and were cultured in DMEM F12 medium supplemented with 10% (v/v) Fetal bovine serum (FBS), 100 µg/ml streptomycin and 100 U/ml penicillin in a humidified atmosphere including 5% CO_2_ at 37 ^°^C and passaged at 80% confluence. 

***Cell viability assay***

HUVEC cells were cultured in 96-well microplates at a density of 5000 cells/well. After they adhered to the substrate and entered a logarithmic growth phase, to investigate the effect of A-MG on HUVEC cell survival, cells were treated with A-MG (1.25-10 µM) for three days. Also, to induce high glucose conditions, concentrations of 5, 10, 20, 30, 40, 50, and 60 mM of D-glucose were used for 72 hr (12, 27). Afterward, cell viability was determined by 3-(4, 5-dimethylthiazol-2-yl)-2,5-diphenyl tetrazolium bromide (MTT) assay. Subsequently, to examine the impact of A-MG on high glucose conditions, HUVEC cells were cultured in 96-well microplates and incubated with A-MG (2.5 μM) (non-toxic concentration which was obtained based on MTT assay) and D-glucose 60 mM. After 72 hr, the cells were treated with MTT reagent (0.5 mg/ml) for 4 hr in an incubator. Then, the upper layer of the medium was removed and the purple formazan product was dissolved in 100 ml dimethyl sulfoxide (DMSO). Absorbance was calculated at 545 nm (630 nm as a reference) using a microplate reader (Start Fax-2100, UK)(28). Cell viability was reported as a percentage of the value in control cultures.

***ROS assay***

HUVEC cells were seeded in 96-well microplates at a density of 5×10^3^ cells/well and treated as explained by MTT assay. DCF-DA reagent is used to measure intracellular ROS. DCF-DA is a non-polar compound that easily passes the cell membrane and is hydrolyzed by esterases to a non-fluorescent derivative (dichlorofluorescin (DCFH)). DCFH in the presence of ROS changed to DCF which is a high fluorescent agent. Finally, cells were incubated with DCF-DA (10 µM) for 30 min at 37 ^°^C and kept away from light. Then cells were washed twice with PBS. The fluorescence intensity was calculated at 485 nm and 527 nm by a microplate reader (29).

***NO assay***

NO production is measured by the Griess reagent. In this reaction, nitric oxide is converted to nitrite and then to nitrous acid in an acidic environment. Subsequently, nitrous acid reacts with sulfanilamide and forms a diazonium salt which reacts with ethylenediamine. Finally, azo dye is formed and absorbance is recorded at 540 nm. Different concentrations of standard samples (sodium nitrite) were prepared and standard curves were drawn. Then, based on the obtained standard curve, the NO content of the samples was calculated (30, 31).

For this purpose, 5×10^5^ HUVEC cells were counted and transferred to t25 flasks. After adding the desired concentrations of A-MG and glucose to each flask and also after 72 hr, the cells were dissociated by 2 ml trypsin (0.25%). Then, trypsin was neutralized by 4 ml DMEM F12 medium and centrifuged at 1100 rpm for 5 min. The supernatant of the cell plate was discarded, mixed by pipetting up and down with 1 ml PBS, and centrifuged again. PBS was poured and 150 µl of homogeneous buffer containing 2ME was added into the cell plate placed on ice, and after pipetting up and down, the samples were transferred to 1.5 ml microtubes. Fifty microliters of cell culture supernatant with an equal volume of Griess reagent were mixed and incubated at room temperature for 10 min and a microplate reader was used to measure the absorbance at 540 nm.

***Western blot***

HUVEC cells (5×10^5^) were seeded into t25 flasks and then incubated as formerly explained. On the last day, cells of each flask were dissociated using 2 ml of trypsin (0.25%). Then, trypsin was neutralized by 4 ml DMEM F12 medium, and cells were centrifuged at 1100 rpm/5 min. Then, a lysis buffer containing 10 mM β-glycerophosphate, 1 mM Na_3_VO_4_, Tris-HCl 50 mM (pH 7.4), 10 mM sodium azide, 1 mM Na_3_VO_4_, 1.0 mM phenylmethylsulfonyl fluoride, 0.2% W/V sodium deoxycholate, 2 mM EDTA, 2 mM EGTA, and protease inhibitor cocktail (Sigma Aldrich, USA) was added to the samples. After that, the Bradford assay kit (BioRad, USA) was used for the measurement of protein concentration. 2× sodium dodecyl sulfate (SDS) blue buffer was added to the samples with an equal volume (1:1 V/V) and boiled for 5 min at 95 ^°^C, aliquoted in 0.2 ml microtubes, and kept in the -80 ^°^C freezer. Each sample was loaded on an SDS-PAGE gel (12%) and electrophoresed with 120 volts. Then, proteins were transferred to a polyvinylidene fluoride (PVDF) membrane (BioRad, USA) and the membranes were blocked with skimmed milk (free from dietary fat) for 3 hr. Subsequently, they were washed with TBST (Tris-Buffered Saline and Tween 20) three times and incubated for 120 min on a rocker with rabbit polyclonal anti- SIRT1 (Cell Signaling #2310, 1: 1000). Also, other membranes were incubated with rabbit antibody against Beclin-1 (Cell Signaling, #3495, 1:1000) and rabbit antibody against LC3 II/I (Cell Signaling, #12741, 1:1000) antibody overnight (18 hr) at 4 ^°^C and washed three times with TBST. Then, membranes were incubated with rabbit horseradish peroxidase-conjugate anti-rabbit IgG (Cell Signaling #7074, 1:3000) for 1.5 hr. Protein bands appeared with enhanced chemiluminescence (ECL) and normalized against β-actin as a control protein. Alliance 4.7 Gel doc (UK) and UV Tec software (UK) were utilized for integrating optical densities and densitometric analysis of bands, respectively.

**Figure 1:**
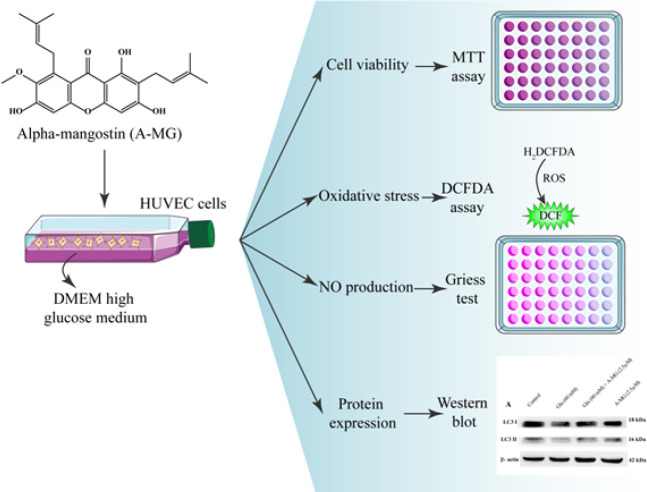
Schematic description of methods used in the study of effect of A-MG on high glucose-induced damage on HUVECs

Schematic description of methods used in this study has been presented in Figure 1.

***Statistical analysis***

For statistical analysis, GraphPad Prism 8 (GraphPad Prism Software Inc., San Diego, CA, USA) was used. Results were shown as mean ± SD in the tests. Statistical comparisons in the mentioned tests were done using one-way ANOVA followed by the Tukey-Kramer test. *P*-values less than 0.05 were considered to be statistically significant. 

## Results

***Effect of different concentrations of glucose on HUVEC***

**Figure 2:**
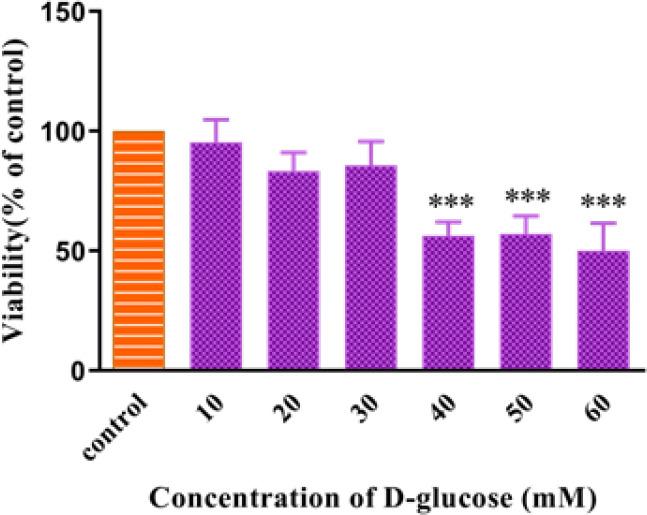
Effect of high glucose concentration on the viability of HUVECs for 72 hr

HUVEC cells were incubated with different concentrations (5-60 mM) of glucose for 72 hr, then performed MTT assay. As shown in Figure 2, at concentrations of 40, 50, and 60 mM of glucose, the reduction in cell viability was significant compared to the control group (*P*<0.001).

***Effect of A-MG on cell viability in HUVEC***

**Figure 3:**
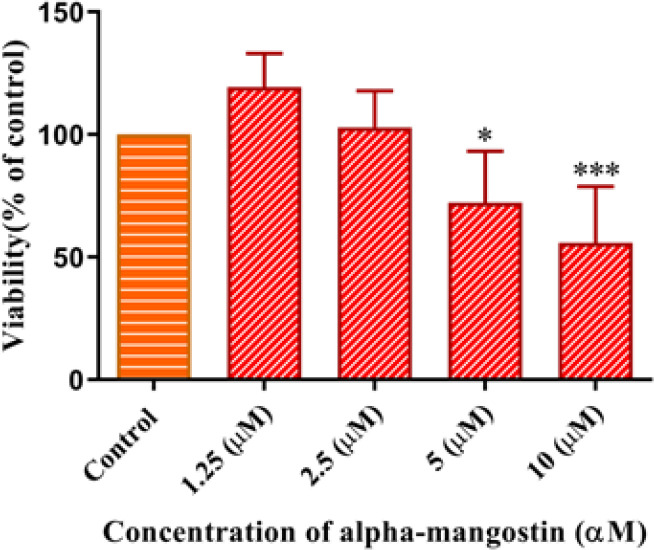
Effect of alpha-mangostin (A-MG) on the viability of HUVECs for 72 hr

To evaluate the effect of A-MG on the viability of HUVEC, these cells were treated with several concentrations (1.25-10 μM) of A-MG for 72 hr. Cell viability was determined by MTT assay. A-MG at concentrations of 5 and 10 μM significantly decreased the cell viability of HUVEC (*P*<0.05 and *P*<0.001, respectively)(Figure 3).

***Effect of A-MG on high glucose-induced cytotoxicity in HUVEC***

**Figure 4:**
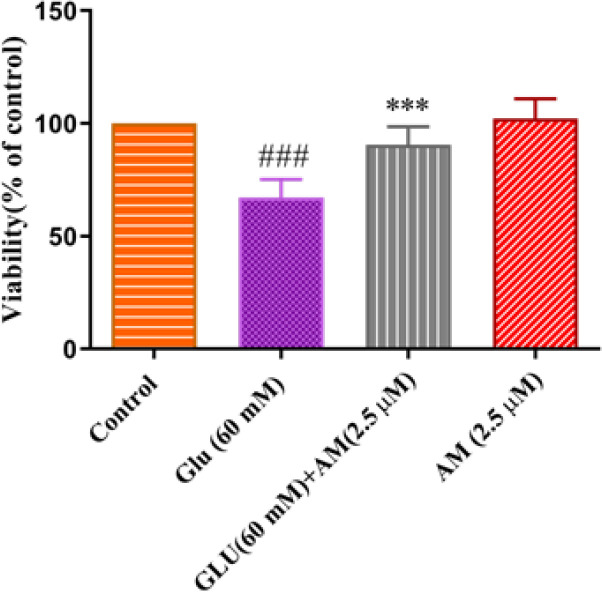
Effect of alpha-mangostin (A-MG) on the viability of HUVECs exposed to high glucose concentration (60 mM) for 72 hr

To evaluate the protective effect of A-MG on high glucose condition toxicity, HUVECs were exposed to A-MG (2.5 μM) and high glucose condition (60 mM) for 72 hr. The next day, cell viability was calculated using the MTT assay. As presented in Figure 4, A-MG at a concentration of 2.5 μM considerably increased cell viability compared to the high glucose condition group (*P*<0.001).

***Effect of A-MG on high glucose-induced ROS in HUVEC***

**Figure 5:**
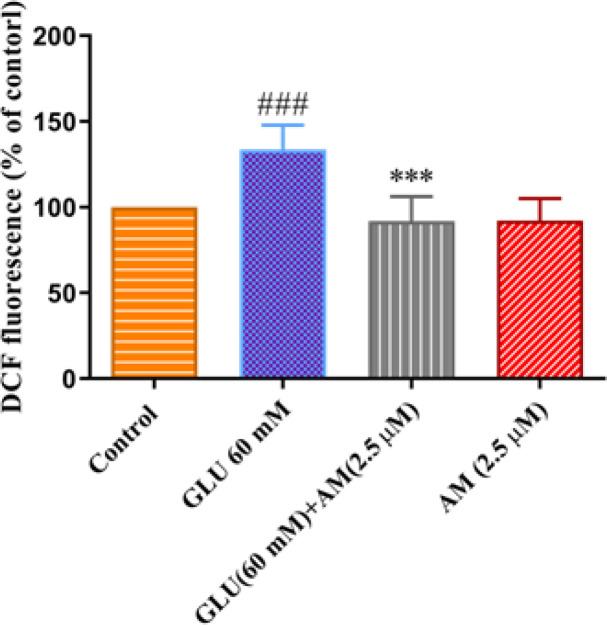
Effect of alpha-mangostin (A-MG)(2.5 µM) on ROS content in HUVECs exposed to high glucose concentration (60 mM) for 72 hr

HUVECs were exposed to high concentrations of glucose (60 mM) and A-MG (2.5 μM) for 72 hr. As shown in Figure 5, the high glucose condition significantly increased the ROS content in cells in comparison with the control group (*P*<0.001). On the other hand, the results have shown that A-MG significantly reduced ROS content in HUVECs (*P*<0.001).

***Effect of A-MG on high glucose-induced NO in HUVEC***

**Figure 6:**
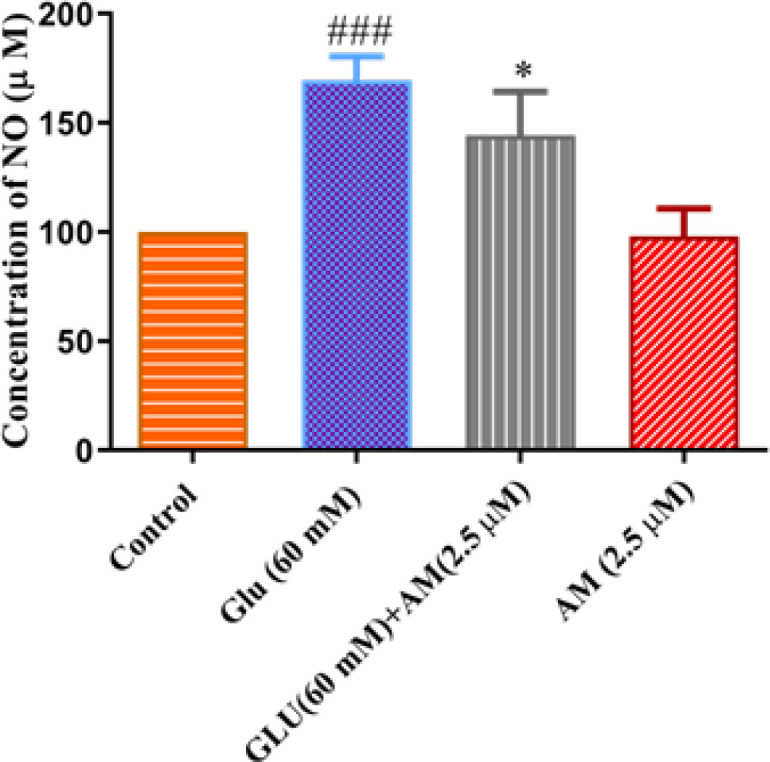
Effect of alpha-mangostin (A-MG)(2.5 µM) on NO content in HUVECs exposed to high glucose concentration (60 mM) for 72 hr

NO content in the high glucose condition group was significantly higher than in the control group (*P*<0.001). A-MG (2.5 μM) significantly reduced NO content in comparison with the high glucose group (*P*<0.05) (Figure 6).

***Effect of A-MG on LC3, Beclin-1, and SIRT 1 protein levels***

**Figure 7:**
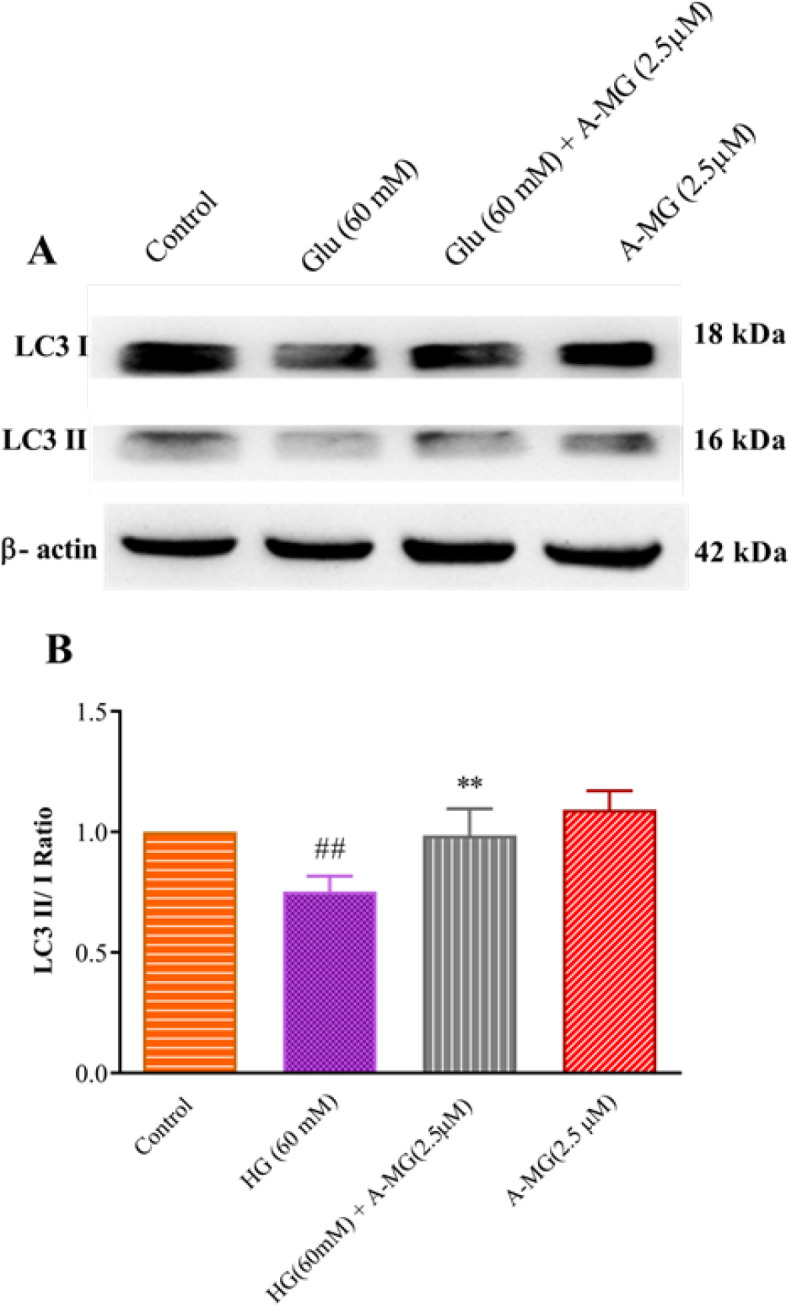
Effect of the alpha-mangostin (A-MG) and high glucose on the protein level of LC3 II/ I ratio in HUVECs

Western blotting was done to measure the protein levels of LC3, Beclin-1, and SIRT 1 in HUVECs. The most effective dose of A-MG (2.5 μM associated with high glucose condition) in previous steps was selected for the western blotting. As shown in Figure 7, in the group exposed to high glucose concentration, the ratio of LC3 II /I was significantly decreased compared to the control group (*P*<0.01). Treatment of A-MG (2.5 μM) notably increased the LC3 II /I ratio in cells (Figure 7).

**Figure 8:**
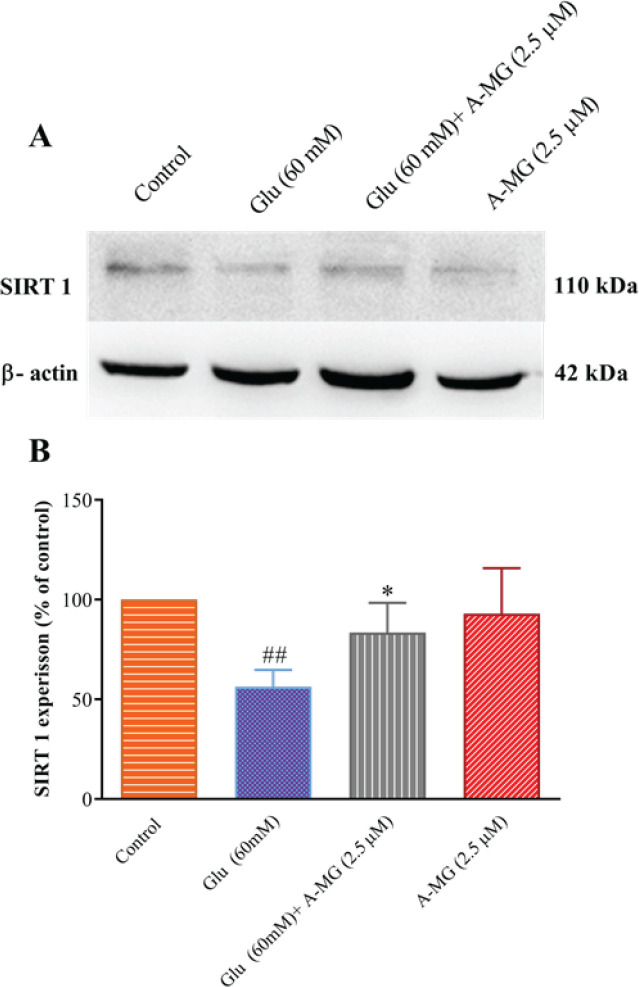
Effect of the alpha-mangostin (A-MG) and high glucose on the protein level of SIRT1 in HUVECs

To evaluate the role of the SIRT1/AMPK pathway in A-MG protection, the SIRT1 amount was measured in HUVECs. According to Figure 8, the high glucose condition significantly decreased SIRT1 in comparison with the control group (*P*<0.01). In contrast, A-MG (2.5 μM) co-treatment statistically increased SIRT1 content in HUVECs (*P*<0.05).

**Figure 9:**
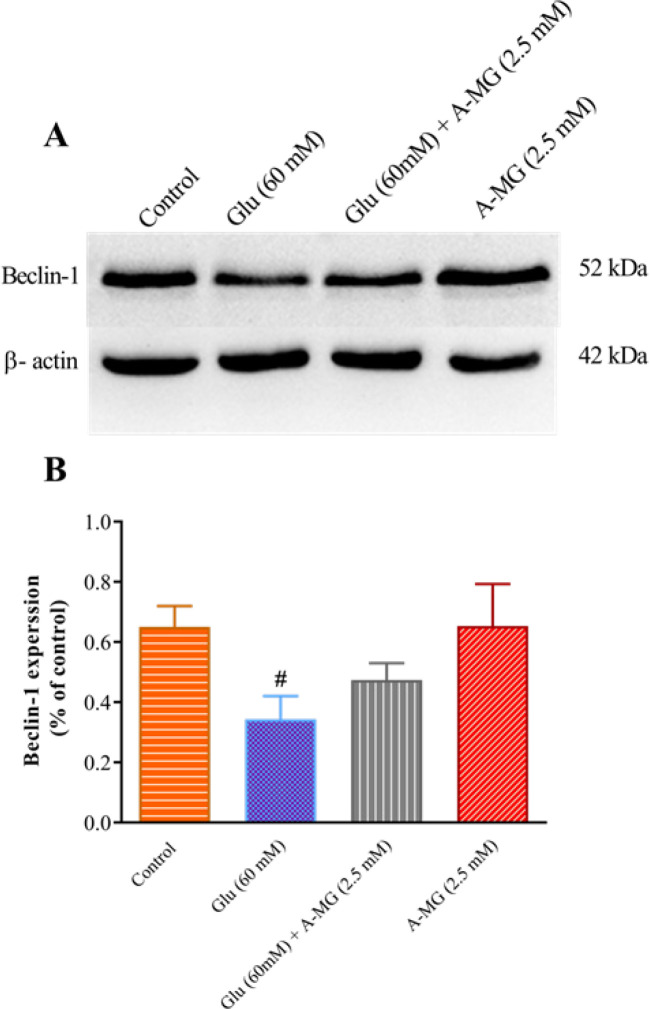
Effect of alpha-mangostin (A-MG) and high glucose on the protein level of beclin1 in HUVECs

As indicated in Figure 9, the high glucose condition considerably reduced beclin1 content in comparison with the control group (*P*<0.05). A-MG could elevate the level of beclin1 but this elevation was not statistically significant (Figure 9).

NO:nitric oxide; ROS: reactive oxygen species; DCFDA: dichlorodihydrofluorescein diacetate; MTT: 3-(4,5-dimethylthiazol-2-yl)-2,5-diphenyl-2H-tetrazolium bromide

One way-ANOVA test and Tukey-Kramer post-test were used to check the statistical difference. Data are presented as mean±SD (n=4). ***P<0.001compared to control group

One way-ANOVA test and Tukey-Kramer post-test were used to check the statistical difference. Data are presented as mean±SD (n=4). *P<0.05 and ***P<0.001compared to the control group

One way-ANOVA test and Tukey-Kramer post-test were used to check the statistical difference. Data are presented as mean±SD (n=4). ^###^*P<*0.001 compared to the control group; ***P<0.001 compared to high glucose group

GLU: glucose; A-MG: alpha-mangostin

One way-ANOVA test and Tukey-Kramer post-test were used to check the statistical difference. Data are presented as mean± SD (n=3). ^###^*P<*0.001 compared to the control group; ***P<0.001 compared to high glucose group

GLU: glucose; A-MG: alpha-mangostin

One way-ANOVA test and Tukey-Kramer post-test were used to check the statistical difference. Data are presented as mean±SD (n=3). ^###^*P<*0.001 compared to the control group; *P<0.05 compared to high glucose group

GLU: glucose; A-MG: alpha-mangostin; NO: nitric oxide

(A) blots and (B) bars showing the densitometry analysis of western blot for LC3 II/ I ratio. One way-ANOVA test and Tukey-Kramer post-test were used to check the statistical difference. Data are presented as mean±SD (n=4). ^##^*P<*0.01 compared to the control group; **P<0.01 to high glucose group

GLU: glucose; A-MG: alpha-mangostin; HG: high glucose

(A) blots and (B) bars showing the densitometry analysis of western blot for SIRT1. One way-ANOVA test and Tukey-Kramer post-test were used to check the statistical difference. Data are presented as mean±SD (n=4). ^##^*P<*0.01 compared to the control group; **P<0.01 to high glucose group

GLU: glucose; A-MG: alpha-mangostin

(A) blots and (B) bars showing the densitometry analysis of western blot for beclin1. One way-ANOVA test and Tukey-Kramer post-test were used to check the statistical difference. Data are presented as mean±SD (n=4). #*P<*0.05 compared to the control group

GLU: glucose; A-MG: alpha-mangostin

## Discussion

A normal regulatory procedure for growth, proliferation, and metabolism in biological systems is autophagy which allows cells to survive in nutritional starvation. Furthermore, autophagy in damaged cells contributed to various diseases such as diabetes, stroke, and cancer (32, 33). A-MG is the most plentiful xanthone in the mangosteen fruits that is used in traditional Chinese medicine for the remedy of chronic ulcers, dysentery, diarrhea, and infected wounds. The protective effect of A-MG on the brain, heart, kidney, and retina tissues against free radical damage has been proven (23, 34).

In the present study, we demonstrated that A-MG at a concentration of 2.5 μM has protective effects under high glucose conditions (60 mM, for three days) through increasing HUVEC viability percentage. Decreased ROS and NO generation were also observed in cells treated with high glucose concentration via A-MG.

The ROS level at common physiological conditions is balanced between ROS production and antioxidant capacity. Nevertheless, this balance can be broken under certain pathological situations and lead to ROS accumulation (35). It was reported that A-MG at concentrations of less than 5 μM has antioxidant activity and elevated catalase activity, glutathione peroxidase activity, and content of GSH in A549 cells but at concentrations of 5 μM and 10 μM antioxidant activity is reversed (36). Similarly, our data showed that A-MG at concentrations of 1.25 and 2.5 μM did not change cell viability but reduced cell viability at higher concentrations. Fang *et al.* reported that A-MG not only increased glutathione (GSH) content, glutathione peroxidase activity, and superoxide dismutase activity but also decreased malondialdehyde (MDA) generation induced by H_2_O_2_ (200 μM) in ARPE-19 cells (23). Also, A-MG (100 mg/kg, intraperitoneally) considerably reduced levels of MDA and NO in arthritic rats (37). In agreement with these studies, our results showed that A-MG (2.5 μM) reduced the level of ROS and NO content in HUVEC cells. As mentioned, A-MG is a xanthan and has several hydroxyl groups. The free radical-scavenging activity of these compounds is related to interaction with a free radical chain of oxidation by donating hydrogen from the phenolic hydroxyl groups. Thus, the free radical chain is broken and lipid peroxidation prevented (38).

Autophagy-related genes encode some special proteins that regulate the autophagy process (17). Accumulating evidence indicated that protein expression of LC3, SIRT 1, and beclin 1 was inhibited under high concentrations of glucose (39-41). Converting LC3-I to LC3-II is a critical marker of the early step of autophagy and is fundamental for autophagosome biogenesis (17). Beclin 1 plays a key role in the formation of autophagosomes through interaction with Vps34 (a class III-type phosphoinositide 3-kinase). Beclin 1-Vps34 complex has a kinase activity feature and facilitates lipid cargo recruitment, membrane extension, and autophagosome maturation (42). A research project reported high glucose stress not only reduced the LC3-II/I ratio but also increased the level of P62 protein in cardiac cells (18). The result of Qu *et al*. revealed that high concentration of glucose decreased autophagosome formation, proliferative activity, and expression of LC3 and Beclin 1 in both RSC96 cells and primary rat Schwann cells (41). A study reported that high glucose condition (30 mM) reduced LC3 II/LC3 I ratio and Beclin 1, elevated levels of oxidative stress markers, and decreased GSH content in HUVEC cells (17). High glucose conditions not only suppressed autophagic genes and proteins such as Beclin-1 and LC3-II/LC3-I ratio but also promoted apoptosis by increased Bax levels and decreased Bcl-2 levels (43). Our results were consistent with the above studies and A-MG reversed high glucose-induced damage by reduction of beclin 1 and LC3 II/LC3 I ratio expression in HUVECs. Contrary to the results of this study, the protein levels of beclin 1 and LC3 II was increased under high glucose condition in podocytes in a time-dependent manner (4). Albeit, Xin *et al*. reported beclin-1 and LC3BII elevated at 2 hr and 24 hr of exposure in the high concentration of glucose but they reduced at 48 hr and 72 hr of exposure (44).

SIRT1, as a member of the class III histone/protein deacetylase family, can regulate the metabolism of lipids and glucose by its deacetylase function (45). SIRT1 conjugated to various ATG proteins including ATG5, ATG7, and ATG8, and prevented autophagy. SIRT1 absence significantly enhanced the acetylation of these autophagic proteins that led to the initiation of the autophagy procedure (46, 47). It was reported that high glucose conditions decreased SIRT1 expression and increased acetylation of p53 protein in corneas from Ins2^Akita/+^ mice and primary human corneal epithelial cells that lead to cell damage (45). On the other hand, SIRT1 is expressed in the blood vessels during growth and controls angiogenesis in endothelial cells by FoxO1 deacetylation (39, 48). Researchers suggested down-regulation of SIRT1 could lead to the reduction of the amount of endothelial progenitor cells under high concentrations of glucose (39). Reduction of new blood vessel growth and endothelial dysfunction is observed in both type 1 and type 2 diabetes (49, 50). Also, it was reported that SIRT1 increased insulin sensitivity by the improvement of mitochondrial dysfunction and decreasing MDA and ROS accumulation in C2C12 cells (51). Similarly, the results of western blot analysis indicated A-MG remarkably elevated levels of SIRT1 in HUVECs compared to the high glucose-induced group. 

## Conclusion

The findings of this study showed the protective effects of A-MG against oxidative and nitrosative stress induced by high glucose in HUVECs. Also, A-MG increases the expression of the proteins involved in the autophagy pathway and might be considered an agent for the prevention or treatment of endothelial dysfunction in diabetes.

## Authors’ Contributions

H H and BM R supervised the whole project, conceived the original idea, verified the analytical methods, and checked the whole procedure and paper. K R did the experiments and analyzed the data. F E helped in performing the experiments, analyzing the data, and writing the manuscript. All authors have read and approved the manuscript.

## Conflicts of Interest

The authors declare that we have no conflicts of interest
